# Extract of *Ganoderma formosanum* Mycelium as a Highly Potent Tyrosinase Inhibitor

**DOI:** 10.1038/srep32854

**Published:** 2016-09-09

**Authors:** Kai-Di Hsu, Hong-Jhang Chen, Chi-Shin Wang, Chi-Chin Lum, Shu-Pei Wu, Shin-Ping Lin, Kuan-Chen Cheng

**Affiliations:** 1 Institute of Biotechnology, National Taiwan University, Taipei 10617, Taiwan; 2 Graduate Institute of Food Science Technology, National Taiwan University, Taipei 10617, Taiwan; 3 Department of Plant Pathology and Microbiology, National Taiwan University, Taipei 10617, Taiwan; 4 Department of Medical Research, China Medical University Hospital, China Medical University, Taichung, Taiwan

## Abstract

In this study, the inhibitory effect of *Ganoderma formosanum* mycelium extracts on tyrosinase, the central regulatory enzyme being responsible for cutaneous pigmentation, was investigated in both cell-free and cellular enzymatic systems, as well as in phenotype-based zebrafish model. Bioassay-guided purification indicated that the ethyl acetate fraction of *G. fromosanum* mycelium ethanolic extract (GFE-EA) demonstrated the highest inhibition toward cell-free tyrosinase (IC_50_ = 118.26 ± 13.34 ppm). The secreted and intracellular melanin of B16-F10 cells were reduced by GFE-EA through suppression of tyrosinase activity (IC_50_ = 102.27 ± 9.49 ppm) and its protein expression. Moreover, GFE-EA decreased surface pigmentation level of zebrafish via down-regulation of tyrosinase activity. Most of all, there is no significant difference in morphology and mortality between control and GFE-EA treated groups. Not only does GFE-EA exhibit similar depigmenting efficacy to kojic acid with lower dosage (approximately one-seventh of dose), but show less toxicity to zebrafish. It is worth noting that GFE-EA is extracted from mycelium, which subverts the general concept that mycelium lacks certain bioactivities possessed by fruit bodies. Altogether, it would appear that GFE-EA has great potential for application in the cosmetics industry.

Melanin overproduction and accumulation had been reported to be the causes for cutaneous hyperpigmentation in mammals^1^. Melanin biosynthesis is initiated with tyrosine oxidation by tyrosinase, the enzyme which catalyzes the rate-limiting step for melanogenesis^2^. Upon exposure to ultraviolet radiation, melanin formation plays a critical role in protecting skin from UV-induced DNA damage^3^; however, abnormal melanism such as freckles, solar lentigines and dark spots are regarded as aesthetically unfavorable^4^, having been significantly promoting the development of cosmetic products. Therefore, tyrosinase inhibitors have received great attention in the field of cosmetics due to the amelioration of skin pigmentation disorders. To date, several skin depigmenting agents, such as kojic acid and arbutin, are being sold as commercially available products. Nevertheless, due to the concerns related to adverse effects and long-term effectiveness, novel tyrosinase inhibitors with higher activity, lower toxicity and sufficient penetrative ability are still needed^5^.

Tyrosinase, a glycoprotein located in the membrane of the melanosome^6^, is a common target for depigmenting agents which are intended to alleviate skin pigmentary abnormalities. The two rate-limiting reactions of melanin biosynthesis are initially catalyzed by tyrosinase: the hydroxylation of L-tyrosinase to 3, 4-dihydroxyphenylanlanine (L-DOPA) and the subsequent oxidation of L-DOPA to dopaquinone. Furthermore, dopaquinone is a highly reactive ortho-quinone, which forms melanin by spontaneous polymerization^7^. Further, melanin was transferred to keratinocytes by melanocytes, and each epidermal melanocyte interacts with approximately 36 keratinocytes during melanosome transfer^8^. Interestingly, the number of tyrosinase molecules present in black or white skin color is equal; therefore, the level of tyrosinase activity in melanocytes will regulate cutaneous pigmentation^9^.

In traditional Chinese medicine (TCM), *Ganoderma* spp., commonly known as lingzhi, has recently attracted interest for its free radical scavenging and anti-aging activities^10^,^11^. Three decades ago, *Ganoderma formosanum*, an endemic species of *Ganoderma* in Taiwan, was firstly isolated from Formosan sweet gum (*Liquidambar formosana*) in Taoyuan County, Taiwan^12^. In previous studies, PS-F2, a polysaccharide fraction of *G. formosanum*, is found to have the ability to stimulate macrophage and cytotoxic T cell activation^13^,^14^; however, other bioactivities of *G. formosanum* remain unknown. Nowadays, the cosmetically efficacy of *Ganoderma* spp. have been studied rather than its traditional uses. By many accounts, *Ganoderma lucidum* extracts from fruit bodies exhibited the highest tyrosinase inhibition activity (IC_50_ value 0.32 mg/mL) in comparison with other Basidiomycetes including *Antrodia camphorate, Agaricus brasiliensis*, and *Cordyceps militaris*^15^. Moreover, ergosterol peroxide isolated from *G. lucidum* MeOH extracts inhibited the melanin level of B16 murine melanoma cells by 7.2% at 2 μg/mL^16^. Yet another issue needed to be conquered is the cost of fruit bodies in scale-up production; consequently, submerged fermentation provides a time-saving and economic way for industrial microbial cultivation^17^.

Zebrafish (*Danio rerio*), a small tropical fish, has been shown to be a powerful phenotype-based model for melanogenic regulatory compounds screening due to its rapid pigment formation during embryonic development^5^. One of the most striking features of this system is its high conservation in protein functional domains to their human protein orthologues, for example, substrate-binding regions^18^. Accordingly, it has been recognized as an effectual model organism for medicinal, toxicological and cosmetic researches^19^. Early stage of zebrafish embryos undergo percutaneous absorption of small compounds through skin and gills, while in maturation stage zebrafish from 7 days post fertilization (dpf) absorb small compounds orally^18^. Furthermore, not only can the skin whitening effects of candidate compounds be observed through the melanin distribution on the surface, but the toxicity can also be determined by the heart beating and survival rate^19^,^20^. All things considered, the application of zebrafish larva on percutaneous testing provides a reliable insight into potential depigmentary ingredients discovery.

The specific aim of this study was to discover novel anti-melanogenesis agents from submerged cultivation of *G. fromosanum*. To screen and select candidates of active tyrosinase inhibitors, several fractions isolated from ethanoic extracts of *G. fromosanum* mycelia were investigated through *in vitro* cell-free tyrosinase assay, cell-based and zebrafish phenotype-based platforms.

## Results

### Inhibitory effects of *G. formosanum* extracts on cell-free tyrosinase activity

To screen the potential tyrosinase inhibitors from *G. formosanum* extracts, cell-free tyrosinase was used to be a platform due to its commercial availability^21^. Among the fractions examined, all of them exerted inhibitory effects in a dose-dependent manner (data not shown), and ethyl acetate fraction of *G. fromosanum* ethanolic extract (GFE-EA) exhibited highest inhibition of tyrosinase activity (IC_50_ value was 118.26 ± 13.34 ppm) than other fractions (Table 1). The considerable inhibitory activity on cell-free tyrosinase by GFE-EA suggests that GFE-EA might be a potential candidate for further study.

### Effects of GFE-EA on cell viability

**Figure 1:**
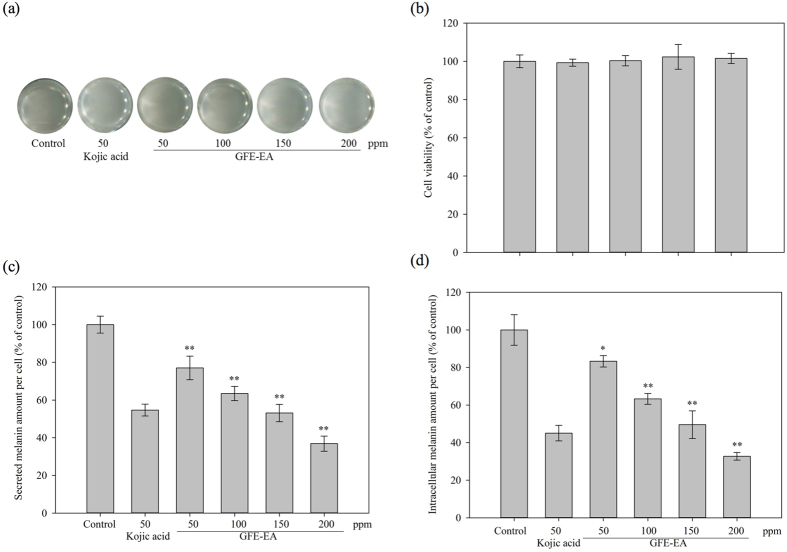
Effects of GFE-EA on melanogenesis in B16-F10 melanoma cells. (**a**) B16-F10 melanoma cells were treated with 50–200 ppm of GFE-EA for 48 hours. (**b**) Cell viability, (**c**) secreted, and (**d**) intracellular melanin amount per cell were examined by methods as described in Materials and Methods. Average data (n = 3) are presented with an error bar of SD (Standard deviation). A value of p < 0.05 (*) or p < 0.01 (**) was performed by Student’s t-test and compared to control group without drug treatment.

To investigate the non-toxicity concentration of GFE-EA, WST-1 assay was applied to B16-F10 melanoma and HaCaT cell lines with various concentrations of GFE-EA for 48 hours. HaCaT keratinocyte cell is a spontaneously immortalized human keratinocyte cell line from adult human skin, which has been widely used for dermatology studies^22^. Therefore, B16-F10 melanoma and HaCaT cells were used to elucidate whether GFE-EA exerts cytotoxic effects. As shown in Fig. 1b, GFE-EA didn’t demonstrate cytotoxicity toward both B16-F10 melanoma and HaCaT cells (Figure S1) with a concentration range of 50–200 ppm, indicating that GFE-EA exerted no cytotoxic at the tested concentrations. Therefore, concentrations below 200 ppm were applied to the following cell-based assay.

### GFE-EA exerts anti-melanogenesis effects on B16-F10 melanoma cells

B16 melanoma cells are well-established model for melanogenic inhibitors discovery as previous studies suggested^2^,^23^,^24^. In our research, the melanin content of B16-F10 melanoma cells without drug treatment was assigned as 100%. Kojic aicd, a famous cosmetics agent, was used as positive control. Besides, the secreted and intracellular melanin amount per cell, rather than total melanin in previous investigations^23^,^25^, were evaluated. In B16-F10 melanoma cells, GFE-EA exerts stronger anti-melanogenesis effects on secreted and intracellular melanin in a dose-dependent manner (Fig. 1a), and the concentration of GFE-EA required for a 50% inhibition (IC_50_) in secreted and intra-cellular melanin were approximately 150 ppm (Fig. 1c,d).

**Figure 2:**
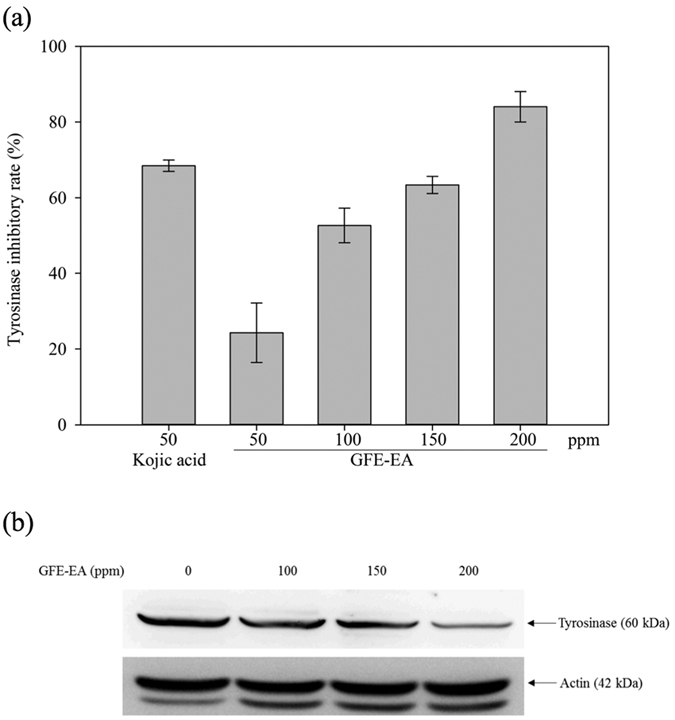
GFE-EA inhibits tyrosinase activity and attenuates the protein level of tyrosinase in B16-F10 melanoma cells. (**a**) For assays of tyrosinase activity, 50 μg of total protein from B16-F10 melanoma cells was incubated with 2 mM L-DOPA, and the level of dopachrome was determined by a photometric method as described in Material and Methods and expressed as percentage of control. (**b**) Whole cell lysates, treated with 100–200 ppm of GFE-EA for 48 hours, were analyzed by western blotting with antibody against tyrosinase. Equal protein loading was confirmed by antibody against β-actin (full-length gels and blots are presented in Supplementary Figure S2).

In addition, as tyrosinase is involved in melanin biosynthesis, measuring cellular tyrosinase activity is pivotal to evaluate the depigmenting mechanism of candidate ingredients. As shown in Fig. 2a, we found GFE-EA inhibits cellular tyrosinase activity in a dose-dependent manner. Besides, the protein level of tyrosinase was also down-regulated by GFE-EA (Fig. 2b). This finding suggested that GFE-EA decreased melanin synthesis by suppressing both tyrosinase activity and protein level in B16-F10 melanoma cells.

### Effects of GFE-EA on melanin synthesis and tyrosinase activity in zebrafish

**Figure 3:**
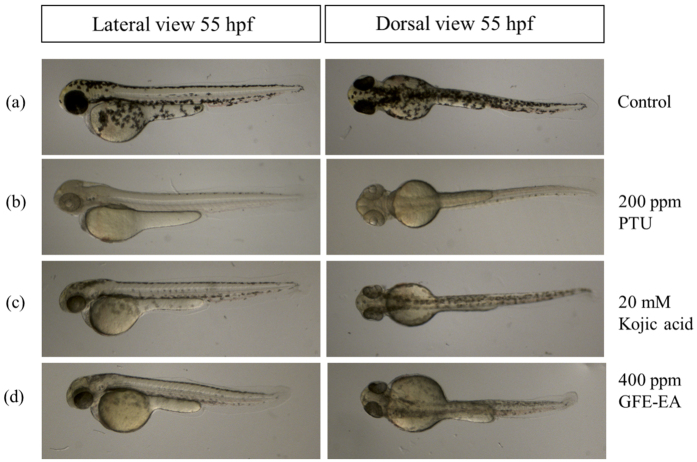
Depigmenting effect of GFE-EA and melanogenic regulators on melanogenesis of zebrafish in an *in vivo* phenotype-based system. Representative images of synchronized zebrafish embryos treated with melanogenic regulators at 55 hpf (48 hours treatment). Depigmenting efficacy of melanogenic regulators on zebrafish were photographed under stereomicroscope at the same magnification. (**a**) Zebrafish embryo without treatment as a control, (**b**) 200 ppm PTU as a standard positive control, (**c**) 20 mM kojic acid, (**d**) 400 ppm GFE-EA.

**Figure 4:**
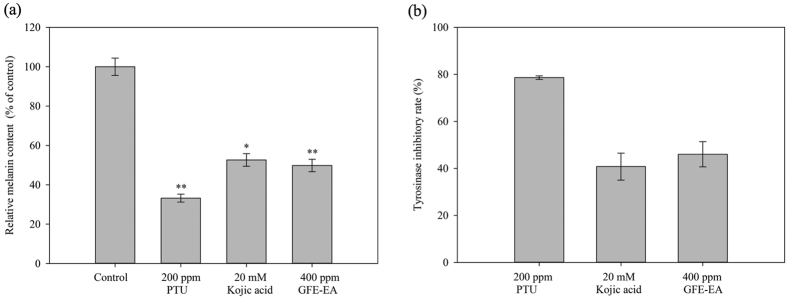
Inhibitory effect of GFE-EA and melanogenic regulators on the melanin and tyrosinase activity in zebrafish. About 70 synchronized embryos were treat with drugs from 7 to 55 hpf. After lysis and centrifugation, melanin was dissolved with 1N NaOH at 100 °C. (**a**) Relative melanin content and (**b**) tyrosinase activity were measured by a photometric method as described in Materials and Methods and expressed as percentage of control. All experiments were repeated as least in triplicate. A value of p < 0.05 (*) or p < 0.01 (**) was performed by Student’s t-test and compared to control group without drug treatment.

To characterize the anti-melanogeneic effects of GFE-EA, total melanin content of whole zebrafish extracts was measured. PTU and kojic acid were positive controls due to their well-known anti-melanogenic activity. PTU, a sulfur-containing tyrosinase inhibitor, is used routinely to block pigmentation in zebrafish^26^ by blocking all tyrosinase-dependent melanogenesis pathway^27^. As expected, both PTU (200 ppm) and kojic acid (20 mM) decreased the melanin content up to 66.8 ± 2.03% and 47.37 ± 3.22%, respectively (Figs 3 and 4a). Meanwhile, there was a significant reduction of melanin content after GFE-EA treatment (p < 0.01). Results show that GFE-EA (400 ppm) exhibited significant inhibition on the body pigmentation up to 51.2 ± 3.11% when compared to control group after 48 hours treatment (Figs 3 and 4a).

To evaluate the hypopigmentary mechanism, we extract total protein from whole zebrafish, followed by incubation with L-DOPA to determine tyrosinase activity. There was evident decrease in tyrosinase activity after treatment with PTU (200 ppm), kojic aicd (20 mM), and GFE-EA (400 ppm). Besides, GFE-EA remarkably decreased tyrosinase activity equal to kojic acid with less dosage (Fig. 4b), suggesting that GFE-EA is a potent inhibitor toward tyrosinase.

### Toxicity assay of GFE-EA in zebrafish

To determine whether GFE-EA exerts toxicity effects on zebrafish, GFE-EA was applied to zebrafish embryos at concentration of 400 ppm for 48 hours treatment and monitored the heart-beating rate, embryo mortality and morphological patterns such as curved trunk, tail bending and relatively small size.

**Figure 5:**
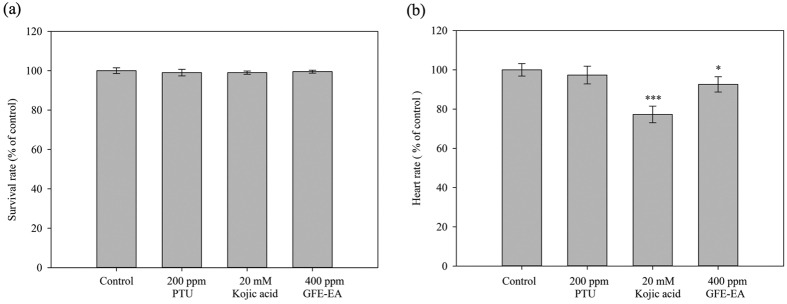
Effects of GFE-EA and melanogenic regulators on the mortality and heart rate in zebrafish. (**a**) The mortality and (**b**) heart rate were determined under a stereomicroscope after 48 hours drug treatment. Each value is presented as mean ± S.D (Standard deviation) from triplicate independent experiments. A value of p < 0.05 (*) or p < 0.001 (***) was performed by Student’s t-test and compared to control group without drug treatment.

In assessing the heart rate, embryos treated with GFE-EA did not demonstrate the apparent difference when compared to control group at 55 hdf (Fig. 5b). Furthermore, morphological malformations were not observed after GFE-EA treatment (Fig. 3a,b). Most importantly, GFE-EA showed no lethal effects on the embryos (Fig. 5a), which implies that GFE-EA did not manifest significant adverse effects to zebrafish. Although kojic acid (20 mM) didn’t result in embryonic lethality (Fig. 5a), it’s noteworthy that kojic acid (20 mM) exhibited a slight disturbance in heart-beating rate (Fig. 5b). Similar phenomenon was also reported in another study^5^.

## Discussion

*Ganoderma* species have been used in China since Before the Common Era (2000 years ago) as nutraceuticals to prevent numerous choric diseases and to improve health, for example, immunodulation, anti-oxidative and reduction of obesity^28^,^29^. To date, the global market size of *Ganoderma* nutraceuticals amounted to 2.5 billion $ (US)^29^. However, little is known about cosmetic effect of *Ganoderma* spp. Therefore, the tyrosinase inhibitory activity of *G. formosanum* was investigated in this study.

*Ganoderma lucidum* had been demonstrated to exhibit significant inhibitory effect on *in-vitro* cell-free tyrosinase activity among tested mushroom including *Antrodia camphorate, Agaricus brasiliensis*, and *Cordyceps militaris*^15^. Nevertheless, its commercialization was limited due to the high production cost of *Gandoerma* fruit bodies. To reduce the cost of cosmetic application, time-saving and economically beneficial submerged fermentation strategy was evaluated. Moreover, *G. formosanum*, an endemic species of *Ganoderma* in Taiwan^12^, was found to possess potent tyrosinase inhibitors in this study. It is worth noting that the IC_50_ of GFE-EA on *in vitro* cell-free tyrosinase activity was 118.26 ± 13.34 ppm, showing superior tyrosinase inhibitory efficacy than *G, lucidum* fruit bodies extracts (320 ppm) in previous study^15^. Most importantly, it subverted the concept that the bioactivity of *Ganoderma* fruit bodies is preferable to mycelium. The results of *in vitro* cell-free tyrosinase (mushroom tyrosinase) assay showed that GFE-EA was a potential tyrosinase inhibitor among the tested fractions; however, due to the structural difference between mushroom (tetramer) and mammalian (monomer)^30^ tyrosinase, the application of GEF-EA on human skin care needs to be confirmed by further studies.

To demonstrate the potential inhibitory effects of GFE-EA on human tyrosinase, the crude murine tyrosinase, an analogous to human tyrosinase, was used to evaluate the feasibility of skin-whitening application in humans^31^. Many studies determined only the intracellular melanin content^25^,^28^, however, a large part of newly formed melanin is secreted into the culture medium and only 12–25% was maintained inside the cell^32^. It is believed that the drugs in pigmentary analysis at an inappropriate dose might result in false-positive result on melanin inhibition due to cell death. Therefore, we treated B16-F10 murine melanoma cells with GFE-EA and investigated its down-regulation effects on secreted melanin, intracellular melanin amount per cell and cellular tyrosinase activity. As expected, kojic acid down-regulated the melanogenesis (Fig. 1c,d). We substantially compare the efficacy of GFE-EA to kojic aicd, and the results showed that hypopigmentary effect of GFE-EA was slightly weaker than those from kojic acid (Fig. 1c,d), and it might be owing to the impurity of crude extract.

To reveal the depigmenting mechanism of GFE-EA, we investigated the effect of GFE-EA on tyrosinase activity. Similar trend of melanin inhibition was also observed in tyrosinase inhibition after GFE-EA treatment (Fig. 3a). Furthermore, tyrosinase protein expression was decreased by GEF-EA (Fig. 3b). Hence, these results suggested that the anti-melanogenesis efficacy of GEF-EA could be accomplished by blocking tyrosinase activity and its protein level.

Granted that the data acquired from both *in vitro* enzymatic and cell-based assay have demonstrated the potential anti-melanogenic activity of GFE-EA, it is still not extrapolated to *in vivo* model directly. The obstacles of traditional *in vivo* animal test, such as mouse model, lie in laborious, uneconomic and compound-consuming^19^. In view of this, alternative model organisms for drug discovery have been developed. Nowadays, zebrafish had been widely recognized as a model organism for drug screening for its fecundity and physiological similar to mammals^33^,^34^. Moreover, it contributes to several aspects of depigmentary agent development, including toxicity, percutaneous and hypopigmentary effects^5^,^19^. Rapid surface pigmentation during zebrafish embryonic development provides phenotype-based evaluation for potential melanogenic inhibitors screening^35^. Skin pigment is fairly visible to the naked eye at approximately 24 hpf^36^. Therefore, zebrafish have been developed as a novel phenotype-based model for screening melanogenic regulatory drugs^5^.

In this study, we successfully adopted zebrafish phenotype-based model. As an illustration in Fig. 3, treatment of the embryos with PTU (200 ppm), kojic acid (20 mM) and GFE-EA (400 ppm) during 7–55 hpf significantly inhibited body pigmentation in developing larvae. For quantification of melanin in zebrafish, image quantification was often adopted in previous study^28^,^37^; nevertheless, more consideration needs to be given to individual difference of body pigmentation in zebrafish. Hence, not only did we isolate the total melanin from whole zebrafish extracts, but it was further normalized with total protein to make the measurement precisely. Among the tested melanogenic regulators, PTU, a standard hypopigmentary agent used in zebrafish lightening model, decreased the melanin content significantly, verifying our experiment design. At the concentration of 400 ppm, GFE-EA demonstrated the same efficacy of transparency as kojic acid (20 mM) in zebrafish embryo (Fig. 3), a well-known skin whitening agent, with approximately one seventh dosage (Fig. 4a). The result was consistent with the trend of tyrosinase inhibitory effects of GFE-EA and kojic acid (Fig. 4b). It is interesting, though, that the result was opposite to that of cell model (Fig. 1c,b). In view of the difference, a possible assumption is that zebrafish absorbed GFE-EA preferably than kojic acid. Above all, *in vivo* zebrafish system provided robust evidence for GFE-EA’s hypopigmentary activity.

Instead of the large number and expensive animals, zebrafish is a promising alternative model for evaluating drug-induced potential toxicity^38^. Although mice were traditionally used for studying the toxicity of cosmetic agent for human application, some drawbacks still exist, including higher consummation of precious compounds, long-term experimental period and unsuitable treatment (oral feeding). Therefore, zebrafish embryos were employed to evaluate whether GFE-EA (400 ppm) exerts toxicity by monitoring morality, heart rate and morphological malformation. There was no obvious difference of appearance, heart-beating and survival rate between GFE-EA and control group (Figs 3 and 5), which implied GFE-EA didn’t show significant adverse effect in zebrafish embryos. It’s noteworthy that kojic acid (20 mM) exhibited disturbance in the heart-beating assay, and similar result was also observed in previous study^5^. As a matter of fact, the use of kojic acid in skin care was banned in Japan due to its carcinogenicity concern^39^.

In summary, GFE-EA was reported as a highly potent tyrosinase inhibitor throughout *in vitro* and *in vivo* screening systems. In addition, the mycelium of *G, formosanum* from submerged fermentation, realizes the scale-up production for commercial application. Furthermore, GFE-EA exhibited same depigmentary effect as kojic acid did with lower dosage and toxicity in zebrafish lightening model. For application in human, further research is needed to validate the depigmentary efficacy and biosafety by applying it on human skin in the future. Moreover, several candidate compounds including ganoderic acids, sterols, polyphenolic compounds and flavonoids were investigated to identify major compound(s) of GFE-EA responsible for depigmenting activity (data not shown). Although which compound(s) contributed to this is still unclear, more detail mechanism of GFE-EA on anti-melanogenesis should be addressed after major compound(s) of GFE-EA identification in our future study. In conclusion, this study suggested that GFE-EA is a potential skin whitening agent and revealed the novel medical application of *Ganoderma* different from traditional uses.

## Materials and Methods

### Submerged mycelial culture of *G. formosanum*

The strain of *G. formosanum* ATCC76537 was purchased from the American Type Culture Collection (Manassass, VA, USA). For the pre-culture, 100 mL of potato dextrose broth (PDB) medium (Acumedia, Lansing, MI, USA) was prepared in a 250 mL flask, and then 8 cm^2^ mycelium from dish culture and was poured at 25 °C on a rotary shaker (120 rpm). After 7 days of cultivation, the seed culture was inoculated (10%, v/v) into a 5 L bioreactor (Firstek, New Taipei, Taiwan) containing 2 L of PDB medium, and followed by a 7-day cultivation at 25 °C with agitation at 120 rpm and aeration at 1 vvm.

### Preparation of crude extracts from *G. formosanum*

The lyophilized biomass of *G. formosanum* obtained from lyophilizer (T10, HCS, New Taipei City, Taiwan) were exhaustively extracted with 95% ethanol and evaporated to dryness under reduced pressure to obtain crude extracts using rotary evaporator (N-1200A, EYELA, Tokyo, Japan). Subsequently, the extracts were dissolved in deionized water and successively partitioned with hexane, ethyl acetate and butanol, respectively.

### Determination of cell-free tyrosinase inhibitory activity

For the determination of cell-free tyrosinase inhibitory activity, the modified method of previous study was used^40^. In brief, 20 μL of cell-free tyrosinase from mushroom (480 units/mL; Sigma, St. Louis, MO, USA) in 20 mM phosphate buffer was mixed with 180 μL of different concentrations of *G. formosanum* extracts. The inhibition rate of tyrosinase activity was calculated as (%) = [1 − (C–D)/(A–B)] × 100. In the calculation, A and B represent the absorbance of vehicle control with and without tyrosinase, respectively; C and D represent the absorbance of the experimental group with and without tyrosinase, respectively.

### Cell culture

B16-F10 melanoma cells (BCRC 60031) were obtained from the Bioresource Collection and Research Center (Hsinchu, Taiwan). HaCaT cell was a gift kindly provided by Dr. R.C. Yu, in the Graduate Institute of Food Science Technology, National Taiwan University (Taipei, Taiwan). Cells were cultured in Dulbecco’s modified Eagle’s medium (DMEM), supplemented with 10% fetal bovine serum (FBS, Hyclone, Logan, UT, USA), 50 U/mL penicillin and 50 μg/mL streptomycin at 37 °C in an incubator containing 5% CO_2_.

### Cell viability assay

Cell viability was determined via WST-1 reagent (Roche, Mannheim, Germany). The B16-F10 melanoma cells were seeded in a 96-well plate at a density of 1 × 10^4^ cells per well. After incubation for 24 hours, the culture medium was replaced with new medium containing various concentrations of *G. formosanum* extracts for 48 hours, then the culture medium was removed and substituted with WST-1 reagent for 30 mins. Cell viability was measured by absorbance at 450 nm using Thermo Multiskan GO (Thermo Scientific, Waltham, MA, USA) and calculated keeping the viability of untreated cells as 100%.

### Measurement of melanin content

The levels of intracellular and secreted melanin were measured as described previously^23^ with a slight modification. The B16-F10 melanoma cells were seeded in a 24-well plate at a density of 5 × 10^4^ cells per well and allowed to attach for 24 hours. The culture medium was removed and replaced with fresh medium containing various concentrations of *G. formosanum* extracts for another 48 hours. For the analysis of secreted melanin, the collected medium was determined by measuring the absorbance at 405 nm and calculated against a known standard curve of synthetic melanin (Sigma, St. Louis, MO, USA). For the measurement of intracellular melanin, the cells had been washed with phosphate-buffered saline and detached with trypsin-EDTA. The cells were collected after centrifugation and lysed in 1 N NaOH at 60 °C for 1 hour. The content of intracellular melanin was measured by absorbance at 405 nm and calculated against a known standard curve of synthetic melanin. Relative melanin content was adjusted by the cell number in the same reaction.

### Determination of cellular tyrosinase activity

Cellular tyrosinase activity in B16-F10 melanoma cells was detected as previous study with slight modification^2^,^41^. In brief, the cells were treated with drugs for 24 hours, which were washed with ice-cold PBS and lysed with 20 mM phosphate buffer containing 1% Triton X-100. After centrifugation for 5 min at 10,000 × *g*, the protein level in supernatant was measured by Bradford assay (Bio-Rad, Richmond, CA, USA). The cellular tyrosinase activity was then examined as follows: 100 μL of the reaction mixture contained 20 mM of phosphate buffer (pH 6.8), 2 mM of L-DOPA, and 50 μg supernatant protein. Following incubation at 37 °C for 1 hour, dopachrome formation was determined by measuring absorbance at 475 nm.

### Western blotting analysis

Western blotting was used to determine the protein expression level of tyrosinase in B16-F10 melanoma cells after GFE-EA treatment for 48 hours. The primary antibody used for the analysis is anti-tyrosinase monoclonal antibody (1:1000, Millipore, Billerica, MA, USA). Monoclonal anti-β-actin antibody (1:5000, Millipore) was used to determine equivalent protein loading in each lane.

### Origin and maintenance of zebrafish

Zebrafish was obtained from the TechComm Zebrafish Core, National Taiwan University (Taipei, Taiwan) and cultured at 28 °C on a 14/10 hour light/dark cycle. Embroys from natural spawning that was cultured in Danieau’s medium [0.45 mM HEPES (pH 7.6), 5.22 mM NaCl, 0.063 mM KCl, 0.054 mM Ca(NO_3_)_2_, 0.036 mM MgSO_4_], with double-distilled water] supplemented with 50 μg/mL of penicillin and 50 μg/mL of streptomycin at 28 °C^42^. All zebrafish maintenance followed the guidelines for the use of laboratory animals and was approved by the Institutional Animal Care and Use Committee at National Taiwan University.

### Phenotype-based evaluation of zebrafish

Phenotype-based evaluation of zebrafish was performed according to the previous study^5^ with slight modification. The collected synchronized zebrafish embryos were arrayed by dropper into dish, one hundred embroys with 4 mL Danieau’s medium. After 7 hpf (hours post fertilization) incubation, the culture medium was replaced with new medium containing the *G. formosanum* extracts (in 1% DMSO) from 7 to 55 hpf (total 48 hours exposure). To evaluate the anti-melanogenesis effects of melanogenic modulators on zebrafish developmental process, the pigmentation of zebrafish was observed under the stereomicroscope at 55 hpf. After deterioration and anesthetization in tricaine methanesulfonate solution (Sigma, St. Louis, MO, USA), embryos were mounted in 2% methyl cellulose on a depression slide and taken images via camera (DFK 23U274, The Image Source, Taipei, Taiwan) under stereomicroscope (Olympus-SZ61, Olympus Optical Co, Tokyo, Japan).

### Tyrosinase activity and melanin contents of zebrafish

Tyrosinase activity and melanin contents assay was performed as described previously with slight modification^5^. In short, about 70 synchronized zebrafish embryos were treated with *G. formosanum* extracts from 7 to 55 hours, and PTU was used as a standard positive control. Total protein was harvested with Tissue PE LB^TM^ lysis buffer (G-BIOSCIENCES, Maryland Heights, MO, USA) supplemented with protease inhibitor and clarified by centrifugation at 4 °C with 10,000 × *g* for 5 mins. The amount of protein was quantified by Bradford assay using bovine serum albumin as a standard (Bio-Rad), then 50 μg of total protein reacted with 1 mM L-3, 4-dihydroxyphenylalanine (L-DOPA) (Sigma) and incubated at 37 °C for 1 hour. The tyrosinase activity was measured by absorbance at 475 nm and calculated as (%) = [1 − (C–D)/(A–B)] × 100. In the calculation, A and B represent the absorbance of vehicle control with and without tyrosinase, respectively; C and D represent the absorbance of the test group with and without tyrosinase, respectively. For determination of relative melanin content, the cell lysate was lysed with 1 N NaOH (the volume of NaOH was adjusted by the protein level among tested reaction) at 100 °C for 1 hour. The level of melanin was measured at the absorbance at 405 nm and calculated against a known standard curve of synthetic melanin.

### Determination of melanogenic inhibitors effects on heart rate

Compound toxicity of melanogenic inhibitor**s** was determined by measuring the heart rate of zebrafish at 55 hpf and compared to vehicle control. Video recording and counting were obtained with camera under stereomicroscope. The obtained results were represented as average heart rate per minute.

### Statistical analysis

All the data in our study were obtained as averages of experiments that were performed at least in triplicate and expressed as means ± SD (Standard deviation). Statistical analysis was performed by Student’s t-test. The significant significance of results was set at p < 0.05 (*), p < 0.01 (**), p < 0.001 (***).

## Additional Information

**How to cite this article**: Hsu, K.-D. *et al*. Extract of *Ganoderma formosanum* Mycelium as a Highly Potent Tyrosinase Inhibitor. *Sci. Rep.* **6**, 32854; doi: 10.1038/srep32854 (2016).

## Supplementary Material

Supplementary Information

## Figures and Tables

**Table 1:** Inhibitory effects of ethanolic extract of *G. formosanym* and its solvent soluble fractions on cell-free tyrosinase.

Samples	Cell-free tyrosinase
	IC_50_^a^ (ppm)
EtOH extract	>1000
*n*-Hexane fraction	>1000
EtOAc fraction	118.26 ± 13.34^b^
*n*-BuOH fraction	>1000
H_2_O fraction	>1000
Kojic acid	23.21 ± 1.32^b^

Cell-free tyrosinase were preincubated with tested samples and subsequent incubated with L-DOPA. To evaluate tyrosinase activity, absorbance was determined at 475 nm and compared with control group without drug treatment. Each value is presented as mean ± S.D (Standard deviation) from triplicate independent experiments.

^a^IC_50_, half maximal inhibitory concentration.

^b^Each value is presented as mean ± S.D (Standard deviation) from triplicate independent experiments.
