# Supplementary material for: Extract of Ganoderma formosanum Mycelium as a Highly Potent Tyrosinase Inhibitor
Source: Sci Rep. 2016 Sep 9;6:32854. doi: 10.1038/srep32854 (PMC5017506; doi:10.1038/srep32854)

**Extract of *Ganoderma formosanum* Mycelium as a Highly Potent Tyrosinase Inhibitor**

Kai-Di Hsu^1^, Hong-Jhang Chen^2^, Chi-Shin Wang^3^, Chi-Chin Lum^2^, Shu-Pei Wu^2^, Shin-Ping Lin^1^, Kuan-Chen Cheng^1, 2, 4^*

1. Institute of Biotechnology, National Taiwan University, Taipei 10617, Taiwan
2. Graduate Institute of Food Science Technology, National Taiwan University, Taipei 10617, Taiwan
3. Department of Plant Pathology and Microbiology, National Taiwan University, Taipei 10617, Taiwan
4. Department of Medical Research, China Medical University Hospital, China Medical University, Taichung, Taiwan.

* Corresponding author. 1, Sec 4, Roosevelt Rd., Taipei 10617 Taiwan;

Tel.: +1 886233661502; fax: +1 886223620847.

E-mail addresses: [kccheng@ntu.edu.tw](mailto:kccheng@ntu.edu.tw) (K.C. Cheng)

**Supplementary information**

**Figure S1. Cytotoxicity effect of GFE-EA on HaCaT cells.** HaCaT cells were treated with various concoction of GFE-EA (50-200 ppm) for 48 hours. Each value is presented as mean ± S.D (Standard deviation) from triplicate independent experiments.

**Figure S2. GFE-EA inhibits tyrosinase activity and attenuates the protein level of tyrosinase in B16-F10 melanoma cells.** Whole cell lysates, treated with 100-200 ppm of GFE-EA for 48 hours, were analyzed by western blotting with antibody against tyrosinase. Equal protein loading was confirmed by antibody against β-actin.

Figure S1.


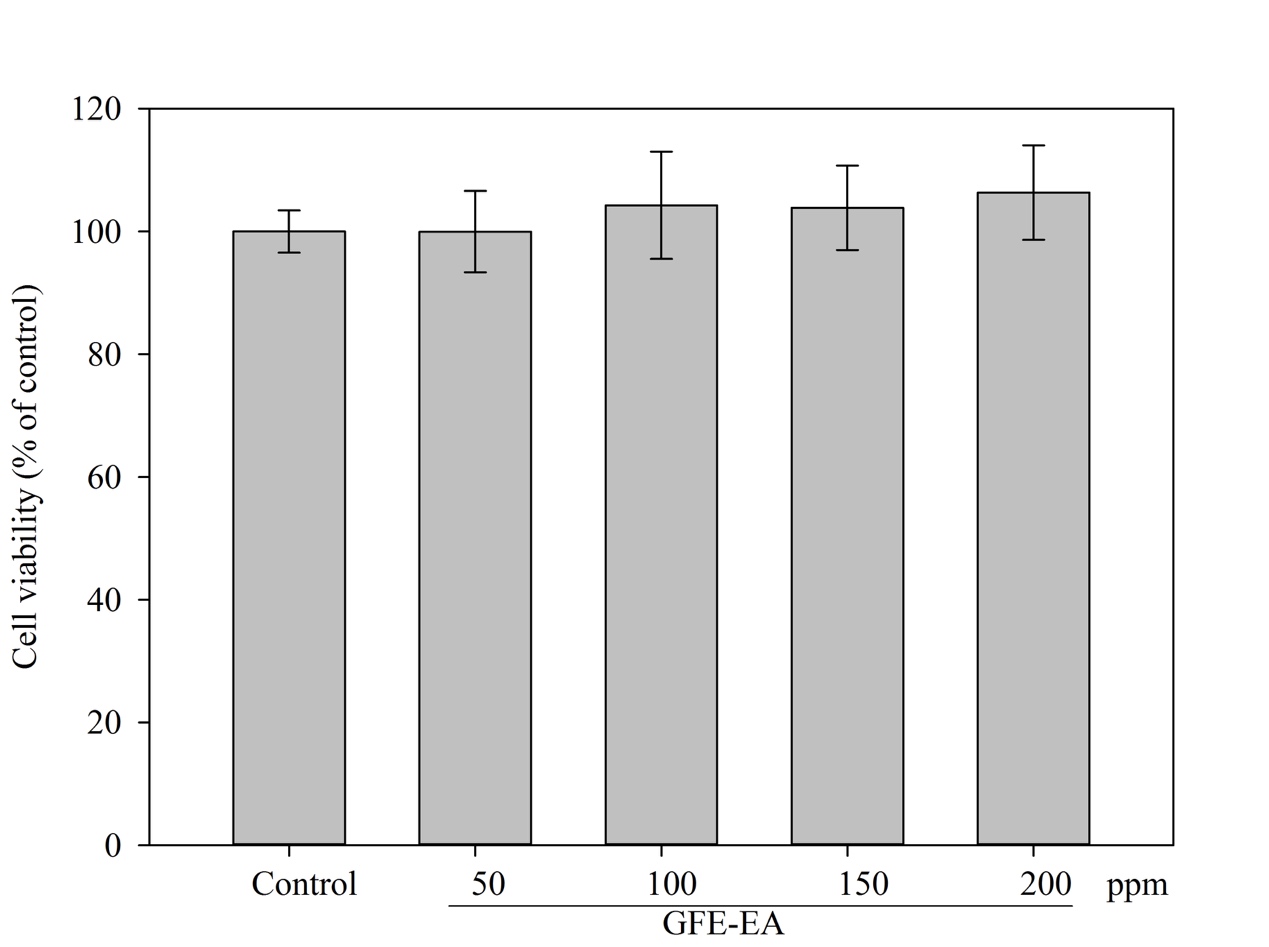


Figure S2.


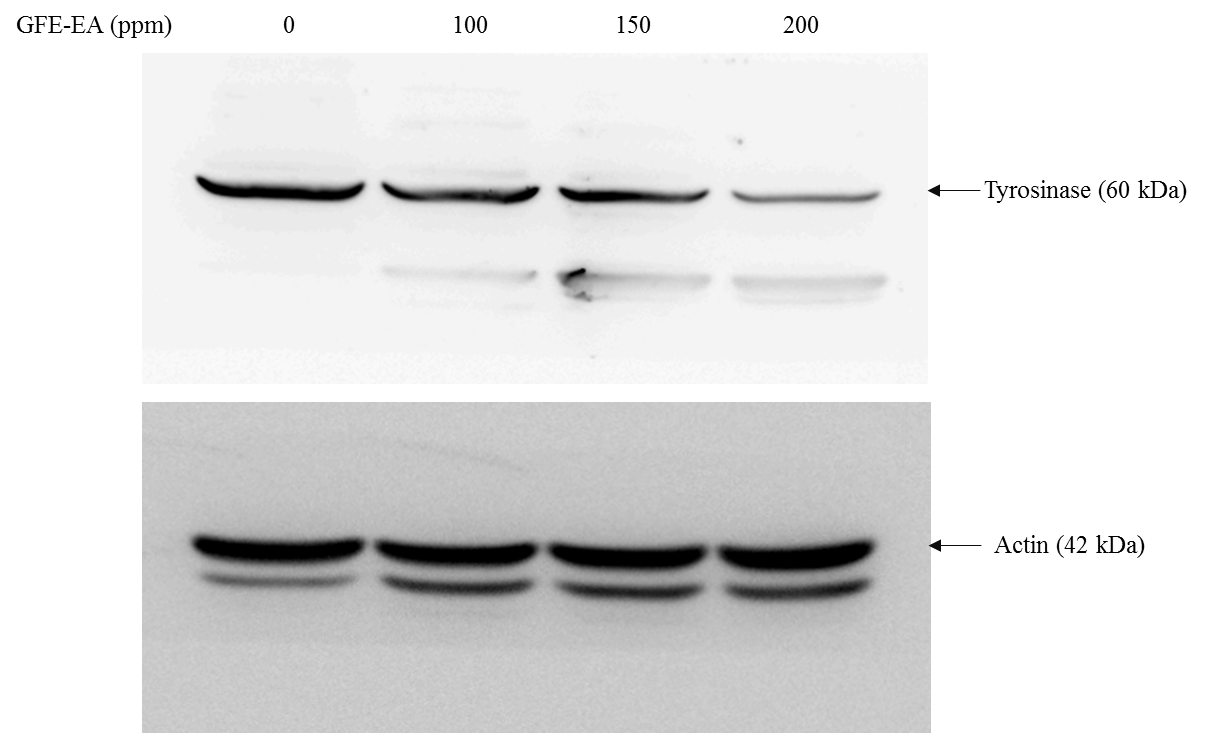

Supplement: Supplementary Information [file srep32854-s1.doc]
